# Bioactive Lipodepsipeptides Produced by Bacteria and Fungi †

**DOI:** 10.3390/ijms232012342

**Published:** 2022-10-15

**Authors:** Antonio Evidente

**Affiliations:** Department of Chemical Sciences, University of Naples Federico II, Complesso Universitario Monte Sant’Angelo, Via Cintia 4, 80126 Naples, Italy; evidente@unina.it; Tel.: +39 081 2539178

**Keywords:** bacteria, fungi, lipodepsipeptides (LPDs), biological activity, structure–activity relationship, potential practical application

## Abstract

Natural products are a vital source for agriculture, medicine, cosmetics and other fields. Lipodepsipeptides (LPDs) are a wide group of natural products distributed among living organisms such as bacteria, fungi, yeasts, virus, insects, plants and marine organisms. They are a group of compounds consisting of a lipid connected to a peptide, which are able to self-assemble into several different structures. They have shown different biological activities such as phytotoxic, antibiotic, antiviral, antiparasitic, antifungal, antibacterial, immunosuppressive, herbicidal, cytotoxic and hemolytic activities. Their biological activities seem to be due to their interactions with the plasma membrane (MP) because they are able to mimic the architecture of the native membranes interacting with their hydrophobic segment. LPDs also have surfactant properties. The review has been focused on the lipodepsipeptides isolated from fungal and bacterial sources, on their biological activity, on the structure–activity relationships of some selected LPD subgroups and on their potential application in agriculture and medicine. The chemical and biological characterization of lipodepsipeptides isolated in the last three decades and findings that resulted from SCI-FINDER research are reported. A critical evaluation of the most recent reviews dealing with the same argument has also been described.

## 1. Introduction

Natural products are the most important source to find compounds with different biological activities and new carbon skeletons, and thus, they have vital importance for agriculture, medicine, cosmetics and other fields. The new naturally occurring compounds also allow the resistance phenomena to be overcome and have potential applications as new eco-friendly solutions in various fields [1,2].

**Figure 1:**
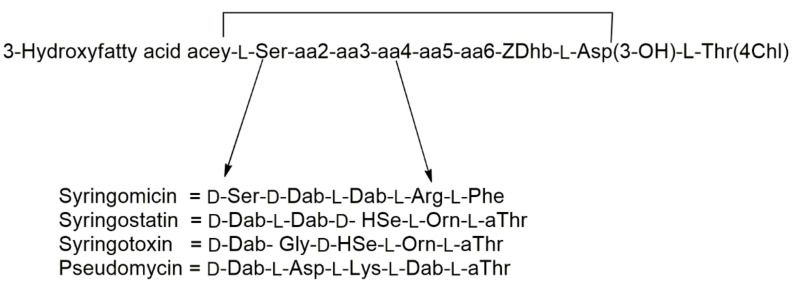
Structures of syringomycin, syringostatin, syringotoxin, and pseudomycin. Abbreviations of non-standard amino acids: Asp(3-OH), 3-hydroxyaspartic acid; Dab, 2,4-diaminobutyric acid; Dhb, 2,3-dehydroaminobutyric acid; Hse, homoserine; Orn, ornithine; Thr(4-Chl), 4-chlorothreonine; aThr, allothreonine.

Among these classes of natural bioactive metabolites, there are the lipodepsipeptides (LPDs), which are biologically active metabolites produced by different bacteria, fungi and marine organisms. They also include cyclic lipodepsipeptides which are subject to many studies due to their important and new biological activities. Cyclic LPDs are constituted by three moieties: (i) a macrocyclic peptide lactone; (ii) a linear peptide; iii) fatty acid. These lipodepsipeptides contain unusual amino acids also with an opposite stereochemistry (D) in respect to that of the common natural spread having a L stereochemistry. They are classified according to their primary structures into two groups (Figure 1). The first group, represented by the phytotoxic cyclic lipodepsinonapeptides constituted by a polar peptide head and a hydrophobic 3-hydroxy fatty acid tail. This group include syringomycins, syringostatins, syringotoxins, and pseudomycins. The four lipodepsinonapeptide subgroups differ in the amino acid sequence between the positions 2 and 6. The 3-hydroxy fatty acyl group is a derivative of either decanoic (syringomycins), or dodecanoic (syringomycins and syringostatins), or tetradecanoic (all four lipodepsinonapeptides), or hexadecanoic (pseudomycins) acid. Some pseudomycins are acylated by either 3,4-dihydroxytetradecanoate or 3,4-dihydroxyhexadecanoate (Figure 1). Furthermore, the three known syringomycins (SRs) only differ themselves by the length of the 3-hydroxy fatty acid moiety, which is either decanoic acid for SRA1, or dodecanoic acid for SRE, or tetradecanoic acid for SRG. Through an amide bond, the carboxylic groups of these acids linked the *N*-terminal serine residue, which in turn is bonded to 4-chlorothreonine at the C terminus through an ester bond generating a macrocyclic lactone ring [3].

**Figure 2:**
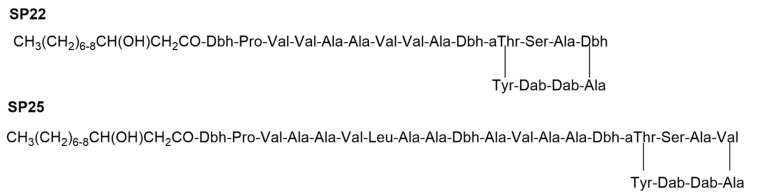
Structures of syringopeptin forms SP22 and SP25. The fatty acid can be either 3-hydroxydecanoic or 3-hydroxydodecanoic acid. Abbreviations of non-standard amino acids: Dab, 2,4-diaminobutyric acid; Dhb, 2,3-dehydroaminobutyric acid; *a*Thr, allothreonine. D-Amino acids are common in both SP22 (13 of 22 residues) and SP25 (15 of 25 residues).

The second group of LPDs, which is represented by phytotoxic syringopeptins (SPs) (Figure 2), was isolated from *Pseudomonas syringae* pv. *syringae* strains [4]. Syringopeptins, different from lipodepsinonapeptides, contain either 22 or 25 amino acids depending on the specific bacterial strain producer. The *N*-terminal amino acid, which is the 2,3-dehydro-2-aminobutyric acid, is acylated by either 3-hydroxydecanoic or 3-hydroxydodecanoic acid. The lactone ring was generated by the ester bond between allothreonine and the C-terminal tyrosine. A high percentage of hydrophobic amino acids are found in the syringopeptin peptide chain, most of which possess a D-configuration [5]. The octapeptide cationic loop formed by a lactone ring, together with the hydrophobic tail, are responsible for their function as a membrane-permeabilizing tool which significantly affected the biological activity [5]. Syringopeptins, which are produced by several strains of *P. syringae* pv. *syringae* differed in the peptide sequences [4,6]. For example, a strain of *P. syringae* pv. *syringae* isolated from laurel produced syringopeptin 25A with phenylalanine as the C-terminal amino acid instead of tyrosine [7]. Strain SC1 of *P. syringae* pv. *syringae* isolated from sugarcane produces a form of SP22 that differs from the previously SP25A for the substitution of Leu residue at amino acid positions 4 and 7 and for the 2-aminodehydropropionic acid (dehydroalanine) at position 9 [8].

Regarding LPDs produced by either bacteria or fungi, there are no references available before 1990. However, lipodepsipedptides are partially referenced in the last three decades in some previously published reviews. In particular, the pharmacological activity of LPDs and their potential as promising lead structures for the development of novel synthetically derived drugs were described by Bionda and Cudic [9]. Some of those compounds are either already marketed (daptomycin 37) or in advanced stages of clinical development (ramoplanin 32) for the treatment of complicated infections caused by multidrug-resistant bacterial strains. The development of new antibiotics based on LPDs is of vital importance because MRSA (methicillin resistant *Staphylococcus aureus*) strains are rapidly increasing, [10,11,12,13,14,15].

LPDs were previously and extensively described in another review based on 135 references regarding their activity as antifungal compounds. In particular, LPDs belonging to the aureobasidin and echinocandin classes were discussed together with aureobasidin A showing in vitro activity against *Candida* and *Cryptococcus* sp. Echinocandins cyclic hexapeptides had been reported as cidal agents inhibiting the synthesis of β-(1,3)-glucan [16].

A table (Table 1) reporting compound names, ring sizes, microbial producers, biological activities, and related references is provided for each LPDs described in the text.

## 2. Source, Isolation, and Biological Activity of Bacterial Lipodepsipeptides

The LPDs reported in this section as well as in the two successive ones are chronologically presented except when different lipodepsipeptides were isolated from the same source or belong to the same subgroup. The data, which are critically described, are based on the direct knowledge of work carried out extensively in the field by the research group of prof. A. Ballio, of whom the author was a pupil working on several fungal phytotoxins. The other data were obtained by SciFinder research on LPDs in the last three decades using appropriate key words. The LPDs as syringotoxins, syringomycins, pseudomycins, syringostatins and syringopeptins, preliminary and briefly described above, fuscopeptins, tolaasins and corpeptins and others, will be described in this section.

The first article on the partially chemical and biological characterization of a syrigomicin was published by Sinden et al. on 1998 [17]. They LPDs produced by *Pseudomonas syringae*, which causes bacterial canker disease of peach trees, were purified using a bioguided purification protocol testing the activity against the model fungus *Geotrichum candidum*. In fact, previously *P. syringae* van Hall, which was pathogenic on stone fruit trees, was described as a producer of a metabolite with a wide antimicrobial activity spectrum. Furthermore, the virulence of several bacterial isolates was correlated with the antimicrobial activity toward *G. candidum*. The metabolites present in the culture’s filtrates were responsible for the observed phytotoxicity [18]. Most of both the antibiotic and phytotoxic activities of this bacterium were due to its ability to produce syringomycins (SR) [19]. The same bacterium also showed the ability to produce phytotoxic syringomycins or syringotoxins but their structures at that time were not determined [20]. 

**Figure 3:**
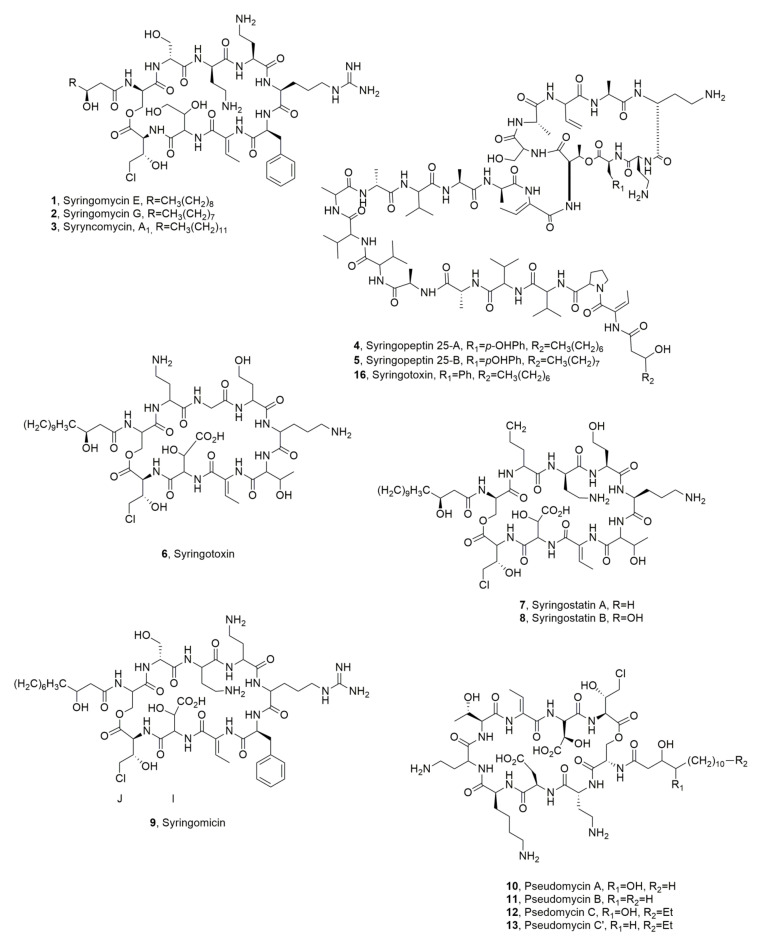
Lipodepsipeptides produced by different strains of *Pseudomonas syringae* pv. *syringae* (**1**–**13** and **16**).

Three SRs, named SR-A_1_, SR-E and SR-G (**1**–**3**, Figure 3) were successively isolated from *P. syringae* pv. *syringae*. The last two LPDs (**2** and **3**) were the most abundant of the mixtures separated by HPLC [21].

Further studies on the culture filtrates of the same bacterium highlighted the presence of other several and more bioactive metabolites. Among them, the main component, as estimated by HPLC analysis, were a lipodepsinonapeptide named syringostatin (ST) and two more hydrophobic lipodepsieicosipentapeptides, named syringopeptins (SP25A and SP25B, **4** and **5**, Figure 3). The biological activities of the metabolite mixtures were different between ST and SPs.

In fact, pure ST exhibited high antifungal and moderate phytotoxic activities, while SP25A was strongly phytotoxic and had a very poor antifungal activity. The biological activity of SP25-B seemed to be very similar to that of SP25A [4].

Syringomycin E and syringopeptins SP25A (**1** and **4**) were also produced by the saprophytic fluorescent strain of *P. syringae* (strain Ml) isolated from wheat. This strain grew in planta without affecting germination or inducing disease symptoms in wheat [22]. 

Syringotoxin (**6**, Figure 3) was produced by *P. syringae* pv. *syringae* isolated from various citrus species. Its structure was determined by Ballio et al. [23]. NMR and molecular dynamic calculation studies allowed for the determination of its conformation in solution. In fact, the evaluation of NOE correlations observed in its ROESY spectrum, recorded in acetonitrile/water, allowed the determination of the nature and number of intramolecular hydrogen bonds and the predominant conformation of ST [24].

Syringostatins A and B (**7** and **8**, Figure 3), two novel phytotoxic LPDs, which also showed antifungal activity, were isolated from *P. syringea* pv. *syringae* SY12 lilac blights in Japan [25].

A new syringomycin (**9**, Figure 1) was isolated from the same bacterial strain SC1, which was obtained from sugar cane in Japan. The bacterial culture filtrates showed antimicrobial activity. LPD **9** differs from syringomycin E in the sequence of units J and I and the α-peptide linkage of unit I [26].

Pseudomycins A, B, C, C’ (**10**–**13**, Figure 3) were isolated together with syringopeptins 25A and 25B (**4** and **5**), from the culture filtrates of *P. syringae* MSU 16H [27] which is a transposon-generated mutant of a wild-type strain. The strain MSU 16H showed a higher ability to protect than the wild-type strain isolated from elms infected with *Ceratocystis ulmi*, the causal agent of Dutch elm disease [28]. The biological activities of pseudomycin A (**10**) were compared to that of syringomycin E (**1**). Compound **10** showed phytotoxicity and activity in vitro and in vivo towards some fundamental processes of plant plasma membrane similar to those of syringomycin E [29]. 

**Figure 4:**
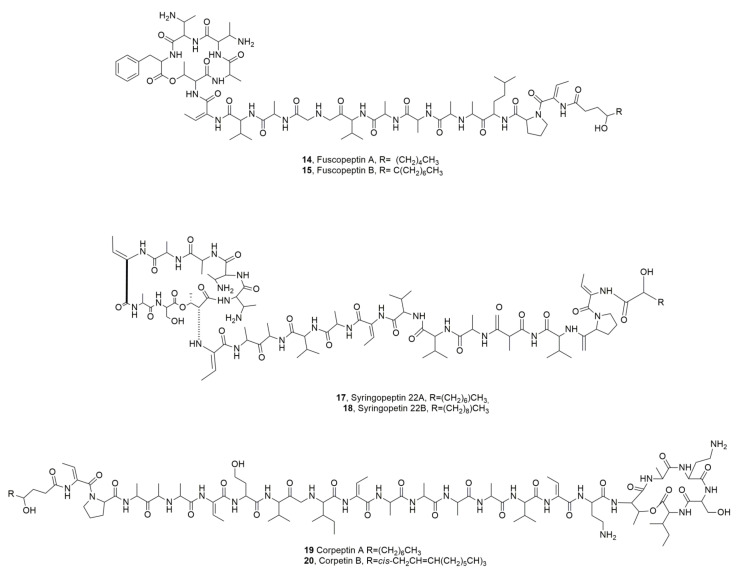
Lipodepsipeptides produced by *Pseudomonas fuscovaginae* (**14** and **15**), *Pseudomonas syringae* pv. *syringae* (**17** and **18**), and *Pseudomonas corrugata* (**19** and **20**).

Two new LPDs were isolated from *Pseudomonas fuscovaginae*, which is the pathogen responsible for the bacterial sheath brown rot in rice and other gramineae such as *Hordeum vulgare*, *Triticum aestivum*, *Avena sativa* and *Zea mays* [30]. The two LPDs that were close to the above described syringopeptins (SPs), were named fuscopeptins A and B (**14** and **15**, Figure 4) Compounds **14** and **15** themselves differ only in the fatty acid residue which is a 3-hydroxyoctanoate and a 3-hydroxydecanoate, respectively. Both LPDs (**14** and **15**) showed phytotoxic and antifungal activity [31]. Their conformation in solution was also obtained by a NOE-NMR study [32]. 

A new syringopeptin (**16**, Figure 3) was purified from culture filtrates of *P. syringae* pv. *syringae*, which was obtained from a twig dieback of Laurel (*Laurus nobilis* L.). LPD **16** is very close to syringopeptin 25A, which differs for the substitution of tyrosine residue as C-terminal of the macrocyclic lactone with a phenyl one. Compound **16** showed phytotoxic activity [7].

Syringopeptins SP22A and SP22B (**17** and **18**, Figure 4) were isolated together with syringomycin SRE, SRG, and SRA1 from *P. syringae* pv. *syringae* strain B301D. Both LPDs **17** and **18** in tobacco protoplast assays, caused lysis of protoplasts at 50 ng/mL and showed potent biosurfactant activity at 0.8 mg/mL. The activities of both LPDS **17** and **18** were very similar to those SRs produced by the same bacterium. These results demonstrated that both LPDs secreted by *P. syringae* pv. *syringae* are cytotoxic to plant cells at nanomolar concentrations and induced necrosis due to the formation of ion channels that are freely permeable to divalent cations [33].

**Figure 5:**
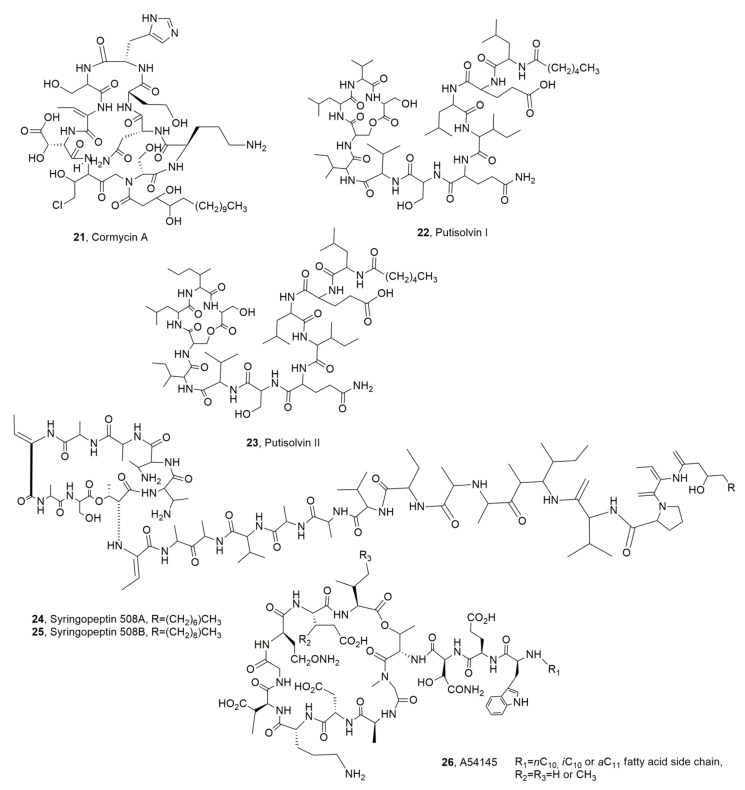
Structures of lipodepsipeptides produced by *Pseudomonas corrugata* (**21**) *Pseudomonas putida* (**22** and **23**) and *Pseudomonas syringae* pv. *lachrymans* (**24** and **25**) and *Streptomyces fradiae* (**26**).

Two LPDs, named corpeptins A and B (**19** ad **20**, Figure 4) were isolated from *Pseudomonas corrugata*, the causal agent of tomato pith necrosis, in which both corpeptins play an important role. The two LPDs themselves differs only for the acyl residue which is a 3-hydroxydecanoyl in LPD **19** and *cis*-3-hydroxy-5-dodecenoyl in LPD **20**. Both LPDs **19** and **20** when assayed in a tobacco leaf induced chlorosis with the LPD **19** being more phytotoxic than compound **20**. Their antimicrobial activities were very similar to those of SPs. LPDs **19** and **20** when tested against the *B. megaterium* showed minimal inhibitory concentrations of 3.75 and 4.20 μM, which are comparable to those for SPs [34], while against *Rhodotorula pilimanae* their activity was negligible [35]. Successively, from the same bacterium another LPD was isolated and named cormycin A (**21**, Figure 5). LPD **21** showed significant, antibiotic phytotoxic and red-blood-cell lysis. Some studies on its structure in solution were also carried out by NOESY NMR experiments and computational calculations [36].

Putisolvins I and II (**22** and **23**, Figure 5) are two biosurfactant LPDs produced by *Pseudomonas putida* strain PCL1445 when the bacterium was isolated from roots of plants and grown on a site polluted with polycyclic aromatic hydrocarbons. LPDs **22** and **23**, which themselves differ in the second amino acid from the C-terminus, which is valine for putisolvin I, and isoleucine for putisolvin II. Both LPDs showed a significant antibiofilm activity [37].

Syringopeptins 508A and 508B (**24** and **25**, Figure 5) were produced by *Pseudomonas syringae* pv. *lachrymans* strain 508, which was isolated in an apple orchard (New York) where it acted in antagonism with *Venturia inequalis*, the causal agent of apple scab [38]. LPDs **24** and **25**, which are very close to syringopeptins 22A and 22B (**17** and **18**), showed growth-inhibition against *Mycobacterium smegmatis*, another gram-positive bacterium, and yeasts [39].

A group of closely related LPDs, named A54145, was produced by *Streptomyces fradiae* [40,41]. A representative structure of this group was reported as compound **26** (Figure 5). This group of LPDs, which is Ca^2+^-dependent, are an emerging class of antibiotics for the treatment of infections caused by gram-positive pathogens. The group A54145 is close to daptomycin, and the calcium-dependent antibiotic (CDA) produced by *Streptomyces roseosporus* and *Streptomyces coelicolor*, respectively. A54145 had antibacterial activity against strains of *Staphylococcus*, *Streptococcus*, *Clostridium* and *Enterococci*. Furthermore, three components of the LPDs mixture (**26**), which were enzymatically deacylated, were re-acylated with different fatty acids and showed antibiotic activity against *S. aureus* and *Streptococcus pyogenes* in mice [42].

**Figure 6:**
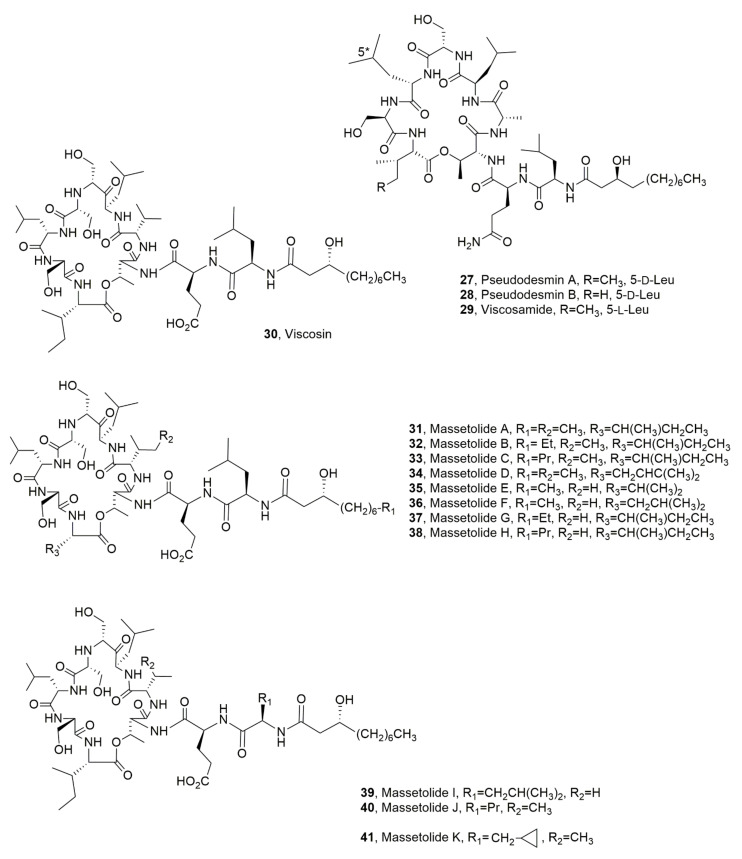
Structures of lipodepsipedtide produced by *Pseudomonas tolaasii* (**27** and **28**), *Pseudomonas fluorescens* and *Pseudomonas libanensis* (**30**), and *P. fluorescens* (**29**–**41**).

Pseudodesmins A and B (**27** and **28**, Figure 6) were isolated from a *Pseudomonas tolaassii*, which was obtained from the mucus layer in the skin of the black belly salamander (*Desmognathus quadramaculatus*). Pseudodesmins A and B are very close to the viscosin group [43]. In this group it needs to be noted that viscosinamide (**29**, Figure 6) differs from the other members in the substitution of glutamic acid at position 2 with a glutamine residue. It can be noted that pseudodesmin A differs from WLIP (White Line Inducing Principle, see below paragraph 3) only for the substitution of D-Glu with a DGln residue. Pseudodesmin A can thus be considered as WLIPamide. Furthermore, LPD **27** differs from viscosinamide, only for the stereochemistry of the Leu at position 5 being D rather than L. Pseudodesmin B (**28**) having L-Val at position 9 differs by at least two substitutions from any previously known viscosin group member. Massetolide E, another member of the same group, had an L-Val9, an L-Leu instead than a D-Leu at position 5 and a D-Glu instead of D-Gln [44]. 

The viscosin group in addition to pseudodesmins A and B and viscosamide (**26**–**29**) and WLIP is constituted by viscosin, massetolides A-H and pseudophomins A and B which all consist of nine amino acid residues, seven of which form the cyclic structure via lactone bond formation between the C-terminus and the side chain of a D-alloThr at position 3. They are produced by several *Pseudomonas* species, including *Pseudomonas viscosa*, *Pseudomonas libanensis*, which produced viscosin [45], different strains of *Pseudomonas fluorescens* which synthesized viscosin and viscosinamide [46,47], the massetolides [48] and the pseudophomins [49] and *Pseudomonas reactans*, which produced WLIP [50].

Viscosin (**30**, Figure 6) was isolated for the first time from *P. viscosa* by Kochi group (1951) [45] during a systematic use of microorganisms able to produce antibiotic metabolites. The LPD **30** named at that time “P-preparation,’’ was obtained as crystals and appeared to be heat stable and soluble in ethanol, methanol, ether, acetone, and alkaline phosphate buffer. Some preliminary investigations assigned an acidic polypeptide structure to **30**, which showed a therapeutic effect in guinea pigs showing tuberculosis symptoms. When more large amounts of this LPD, which was then named viscosin, were prepared, it was possible to demonstrate its antibiotic activity against mycobacteria, its ability to protect embryonated eggs infected with chicken bronchitis virus and to highlight its slight suppressive effect on the infection progress in mice infected with influenza A virus [45]. 

Successively, the LPD **30** was also isolated as a surfactant from a *P. fluorescens* strain and showed a surface tension value of 26.5 mN m^−1^ and a critical micelle concentration of 0.15 mg/mL [51]. 

The structure of viscosin (**30**) was determined for the first time using essentially spectroscopic and degradative chemical methods from Laycock et al. (1991) [46] when it was isolated from a strain of *P. fluorescence* biovars II e IV, which are the causal agents of head rot in broccoli. This is a destructive disease which causes heavy losses in Eastern Canada and other regions of the world. Moreover, its surfactant activity was confirmed. Successively LPD **30** was also isolated as surfactant from *P. libanensis* M9-3. Viscosin had a critical micelle concentration (cmc) of 54 mg L^−1^, its minimum surface tension between air and water at the cmc was 28 mN m^−1^ and it is able to form stable emulsions even at low concentrations (7.5 mg L^−1^). The physicochemical properties recorded for LPD **30** are similar to other biosurfactants such as the well-known rhamnolipid and surfactin. Viscosin, without associated toxicity, also inhibited migration of the metastatic prostate cancer cell line, PC-3M [52]. 

Viscosamide (**29**), above cited, is produced by *P. fluorescens* DR54 and showed biosurfactant and antibiotic properties [47].

Massetolides A-H (**31**–**38**, Figure 6) were isolated together viscosin (**30**) from two *Pseudomonas* sp. strains obtained from a marine alga and a marine tube worm. In particular, LPDs **31**–**34** were isolated from the first organism while LPDs **35**–**38** were produced by the other one [53]. Massetolide A (**31**) and viscosin (**30**) showed in vitro antibiotic activity against *Mycobacterium tuberculosis* and *Mycobacterium avium-intracellulare*. In addition, massetolides I-K (**39**–**41**, Figure 6) were biosynthesized by incorporating nonprotein amino acids. In fact, feeding experiments using L-butyrine and L-norvaline cyclopropylalanine as precursors generated massetolides I, J and K (**39**–**41**) as the main analogues, respectively [53]. Some studies on the biosynthesis of massetolide A were carried out using *P. fluorescens*. LPD **37** is important in the ability of this bacterium to form biofilm [54].

**Figure 7:**
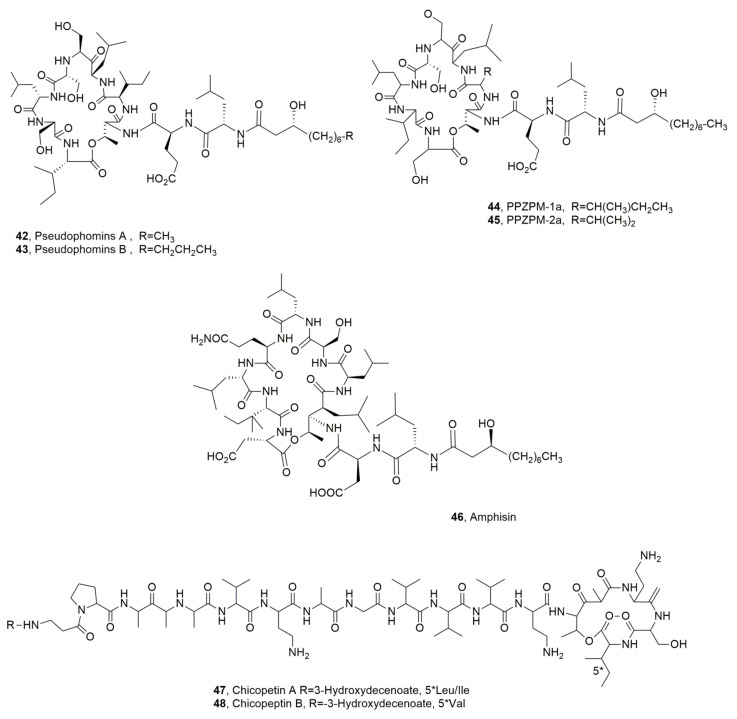
Structures of lipodepsipeptide produced by *Pseudomonas fluorescens* (**42** and **43**), *Pseudomonas* sp. *JX090307* (**44** and **45**), *Pseudomonas* sp. (**46**), and *Pseudomonas cichorii* (**47** and **48**).

Pseudophomins A and B (**42** and **43**, Figure 7) were isolated from *P. fluorescens* strain BRG100, a plant pathogen proposed for weed biocontrol. Pseudophomin B (**43**), which is the main metabolite, exhibited a higher antifungal activity against the phytopathogens *Phoma lingam*, *Leptosphaeria maculans* and *Sclerotinia sclerotiorum* than pseudophomin A. Indeed, pseudophomin A (**42**) was a stronger root germination inhibitor of green foxtail (*Setaria viridis*) than pseudophomin B [49].

PPZPM-1a and PPZPM-2a (**44** and **45**, Figure 7) are the main LPDs among the mixture of more than 30 cyclodepsipetides produced by *Pseudomonas* sp. *JX090307*, which was isolated from hyphae of the phytopathogenic oomycete *Phytophthora alni* spp. *alni* and appeared close to *Pseudomonas orientalis*. The cell extract of *Pseudomonas* sp. *JX090307* showed antifungal activity against *Verticillium dahlia*, some strains of *P. alni* spp. *alni* and different fungal pathogens of forest tree species. The LPDs belonging PPZPM group and thus also compounds **42** and **43**, are constituted by a β-hydroxy fatty acid bonded to a peptide moiety containing 10 amino acids, eight of which generated the macrocyclic lactone, and this later represented the different features with the LPDs viscosin and amphisin groups [55]. Amphisin (**46**, Figure 7) is a lipoundecapeptide produced by *Pseudomonas* sp. strain DSS73, whose structure was determined by X-ray analysis. Amphisin showed biosurfactant and antifungal activity [56]. LPD **46** has diverse applications but is essentially used as a biosurfactant in bioremediation methods to decontaminate dredged harbor sediments as it can increase the bioavailability or mobility of contaminants in an aqueous phase. Amphisin increased the efficacy in releasing polycyclic aromatic hydrocarbons (PAHs) strongly adsorbed to sediments when compared to a synthetic anionic surfactant, [57]. 

*Pseudomonas chicorii* is a pathogen that causes necrotic leaf and stem lesions on several agrarian plants, including lettuce celery, chrysanthemum, tomato, coffee and soybean [58,59,60,61,62]. This bacterium produces phytotoxic metabolite whose role in pathogenesis is not yet demonstrated [63,64,65,66]. Two LPDs, named cichopeptins A and B (**46** and **47**, Figure 7) were isolated from the culture filtrate of *P. chicorii*. LPDs **46** and **47** showed antibacterial activity against *Bacillus megaterium* but not on *Rhodotorula mucilaginosa* and different levels of phytotoxic on the host plant above cited [67].

**Figure 8:**
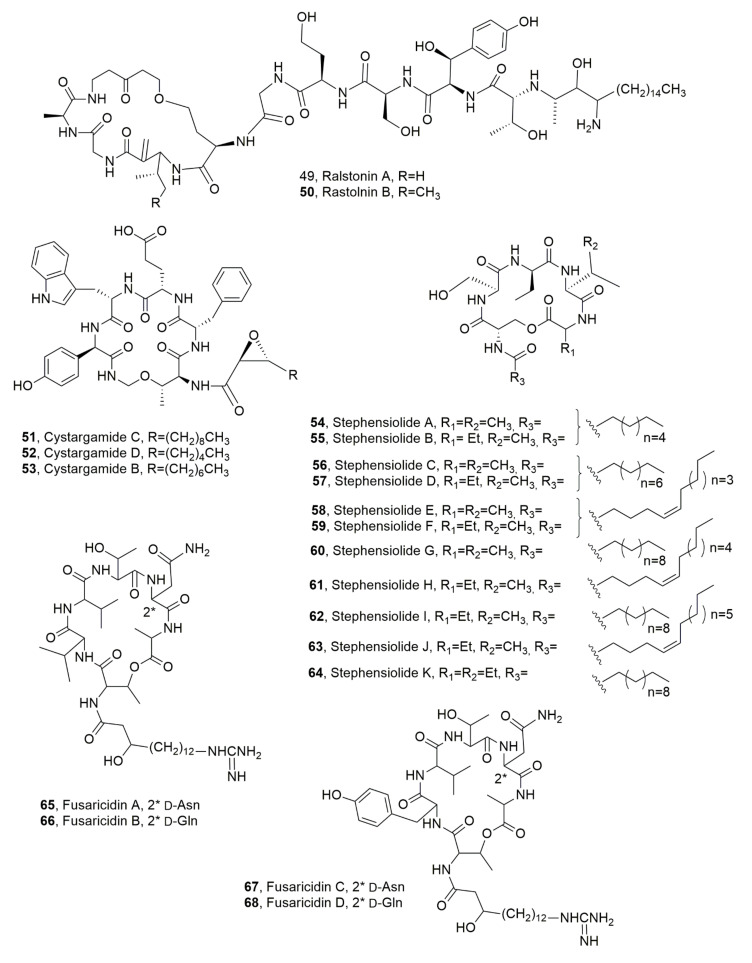
Structures of lipodepsipeptides produced by *Ralstonia solanacearum* (**49** and **50**), *Streptomyces* sp. (**51**–**53**), *Serratia* sp. (**54**–**64**) and *Paenibacillus polymyxa* (**65**–**68**).

Two LPDs, named ralstonins A and B (**49** and **50**, Figure 8) were isolated from *Ralstonia solanacearum*, a β-proteobacterium-inducing lethal disease called “bacterial wilt”, which is observed in more than 200 plant species in tropical, subtropical, and warm temperature regions of the world [68,69,70]. Compounds **49** and **50** are not common among LPDs being constituted of 11 amino acids (containing unique amino acids such as β-hydroxytyrosine and dehydroalanine) and a 3-amino-2-hydroxyoctadecanoic acid. Ralstonins exhibit chlamydospore-inducing activity and moderate phytotoxicity [71].

Cystargamides C and D (**51** and **52**, Figure 8) were isolated, together with previously known cystargamide B (**53**, Figure 8) [72] from a tidal mudflat-derived *Streptomyces* sp. JMS132 collected at Beolgyo, South Korea. The LPDs **51**–**53** showed an antioxidant effect in the DPPH and ABTS assay [73].

Eleven LPDs, named stephensiolides A-K (**54**–**64**, Figure 8) were produced by a *Serratia* sp. found within the midgut and salivary glands of *Anopheles stephensi* mosquitoes. The latter, that contains the *Plasmodium* parasite, has a microbiota that can influence both the vector and the parasite. LPDs **54**–**64** showed antibiotic activity and facilitate bacterial surface motility [74].

Recently a quantitative determination and pharmacokinetic study of fusaridicin A (**65**, Figure 8) in mice plasma and tissues was performed using ultra-high performance liquid chromatography-tandem mass spectrometry [75]. LPD **65** belong to a group of LPDs, named fusaricidins or LI-F antibiotics, which were isolated from *Bacillus polymyxa*, as potential compounds for the development of antibacterial and antifungal agents [9,76,77,78]. In fact, fusaricidins demonstrated strong in vitro antibacterial activity against gram-positive bacteria such as *S. aureus*, *Micrococcus luteus*, and *B. subtilis*. The same group of LPDs showed strong antifungal activities against a broad range of pathogens including *Candida* spp., *Aspergillus* spp., *Penicillium* spp., *Fusarium oxysporum* and *Cryptococcus neoformans* [9,77,78,79]. Fusaricidins A-D (**65**–**68**, Figure 8) were produced by *P. polymyxa* KT-8 strain and showed significant antibiotic activity [77,78]. LPD **64**, among the four isolated fusaricidins, exhibited the strongest activity, towards some gram-positive bacteria e.g., *S. aureus* and pathogenic fungi e.g., *C. neoforman* [77,78,80,81,82,83,84]. 

**Figure 9:**
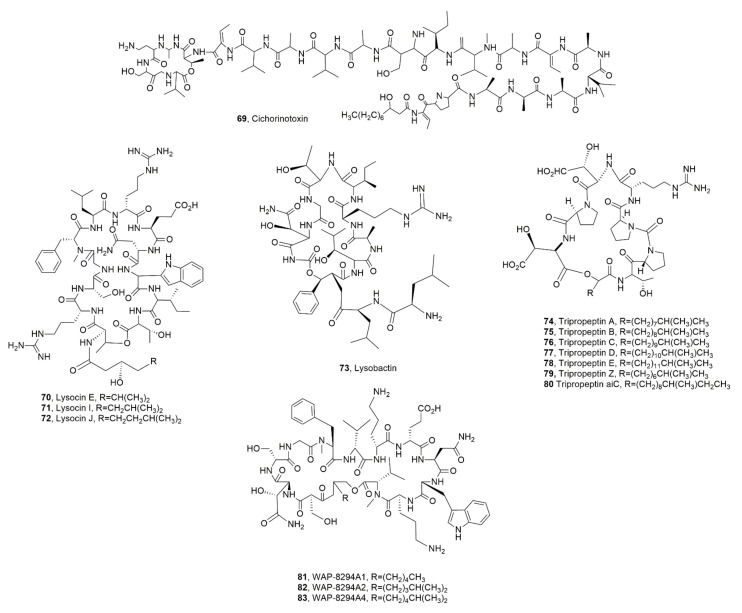
Lipodepsipeptides produced by *Pseudomonas cichorii* (**69**), and *Lysobacter* sp. (**70**–**83**).

A LPD, named cichorinotoxin (**69**, Figure 9) was isolated from *P. cichorii*, which is responsible for varnish spots on lettuces causing serious losses to lettuce production during the summer season in Japan. LPD **69** induce lettuce rot [85].

Lysocin E (**70**, Figure 9) was isolated from *Lysobacter* sp. 3655, and belong to lysocins, which are a group of LPDs showing strong activity against MRSA strains [86,87] associated with a novel mode of action [88]. This antibiotic activity was also shown against *Mycobacterium* spp. in vitro and in silkworm infection models [87]. Successively, lysolicins I and J (**71** and **72**, Figure 9) were isolated together LPD **70** from *Lysobacter enzymogenes*, whose crude extract showed strong antibacterial activity against several gram-positive bacteria [89]. *Lysobacter* are gram-negative bacteria ubiquitously diffused in soil and water [90]. Several *Lysobacter* species produce extracellular lytic enzymes and new bioactive metabolites, which determine their potential both as biocontrol agents and as producers of new drug leads. These metabolites include cyclic peptides, cephem-type β-lactams and polycyclic tetramate macrolactams (PoTeM) [91]. Among LPDs there are lysobactin, the cyclic lipodepsipeptides of tripropeptin family, WAP-8294A family, lysocin family. For some of them including lysocin a total synthesis was realized and mode of action was deeply studied [86,87,92,93,94,95,96,97,98].

Lysobactin (**73**, Figure 9), also known as katanosin B, was produced by *Lysobacter* sp. and its antibiotic activity against aerobic and anaerobic gram-positive bacteria, including *Staphylococci*, *Streptococci*, corynebacteria, clostridia and various other gram-positive anaerobic bacteria, was higher by 2- to 4-folds than that of vancomycin. LPD **73** only had a weak activity against aerobic and anaerobic gram-negative bacteria [99,100].

Tripropetins A-E and Z (**74**–**78** and **79**, Figure 9), were isolated as antimicrobial metabolites from *Lysobacter* sp. strain BMK333-48F3 [92,93,101]. They showed strong activity against MRSA and VRE clinical bacteria strains. In addition, tripropetin C (**76**) blocks the lipid cycle of cell wall biosynthesis by complex formation with undecaprenyl pyrophosphate [102]. Successively, a new LPD, belonging to the same group and named tripropeptin aiC (**80**, Figure 9), was isolated from the same *Lysobacter* strain. LPD **80** showed the same antibiotic activity of the other tripropetins [103].

The LPDs belonging to the WAP-8294A group were isolated from *Lysobacter* sp. WAP-8294 and showed antibiotic activity essentially towards gram-positive bacteria. WAP-8294A2 (**82**, Figure 9) was the major component, while A1, A4, (**81** and **83**, Figure 9) Ax8, Ax9 and Ax13 were the minor ones. These LPDs exhibited antibiotic activity essentially against MRSA strains in vitro [104]. Successively, WAP-8294A1 (**82**) was also isolated from *Lysobacter antibioticus* ATCC 29479 [98].

**Figure 10:**
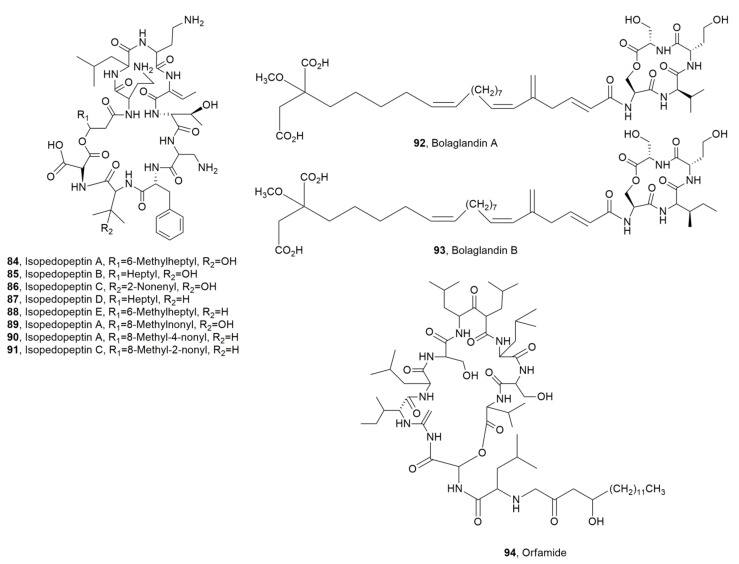
Structure of lipodepsipeptides produced by *Pedobacter cryoconitis* (**84**–**91**), by *Burkholderia gladioli* (**92** and **93**), and by *Pedobacter protegens* (**94**).

Isopedopeptins A-H (**84**–**91**, Figure 10), were isolated from *Pedobacter cryoconitis* strain UP508, which was collected from a soil sample. The LPDs **84**–**91** essentially showed antibiotic activity against gram-negative bacteria. Among them isopedopeptin B (**85**), had not only antibacterial activity, but also cytotoxicity and hemolytic properties. In fact, it exhibited good activity against strains of WHO top-priority gram-negative bacteria, i.e., carbapenem resistant *Acinetobacter baumannii*, *Escherichia coli*, *Pseudomonas aeruginosa*, and against colistin-resistant strains of *A. baumannii*, *E. coli*, and *Klebsiella pneumonia* [105].

Bolagladins A and B (**92** and **93**, Figure 10), were considered two unusual LPDs as they contained citrate-derived fatty acid and a rare dehydro-β-alanine residue. LPDs **92** and **93** were produced by two *Burkholderia gladioli* strains isolated from the lungs of cystic fibrosis patients. LPDs **84** and **85** did not show activity against any of the ESKAPE panel of bacterial pathogens, *Mycobacterium bovis* BCG, or *Candida albicans* [106]. 

Orfamide H (**94**, Figure 10) was isolated from *Pseudomonas protegens* CHA0, which was obtained from tobacco roots growing in suppressive soil [107]. LPD **94** showed antifungal activity inhibiting the appressoria formation of *Magnaporthe oryzae*, the causal agent of the blast severe disease in rice [108].

**Figure 11:**
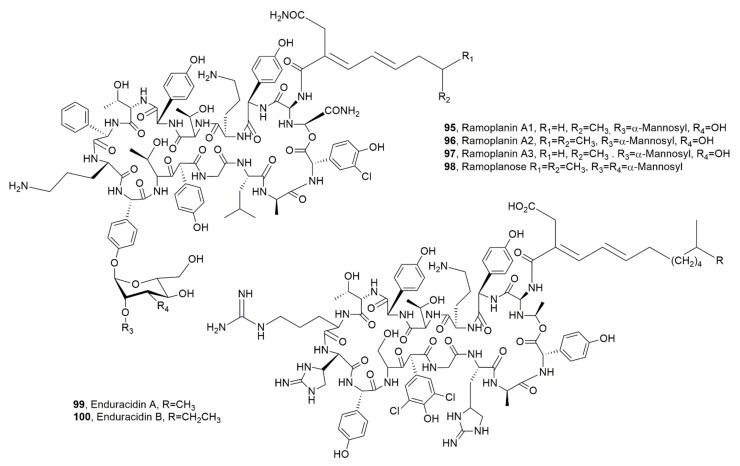
Structures of lipodepsipeptides produced by *Actinoplanes* (**95**–**98**) and *Streptomyces fungicidicus* (**99** and **100**).

Ramoplanins A1, A2, A3 and ramoplanose (**95**–**98**, Figure 11) and the enduracidins A and B (**99** and **100**, Figure 10) were isolated from *Actinoplanes* ATCC 33076 [109,110] and from *Streptomyces fungicidicus* B5477, respectively [111]. Their structures are quite close [112]. Their antibiotic activity is based on their ability to avoid peptidoglycan (PG) cross-link generation inducing bacterial death consequent to osmotic lysis of the cell wall. In particular, LPD ramplanin A2 (**96**) induce membrane depolarization in *S. aureus*, as a complementary mode of action for the disruption of lipid membrane integrity [113]. Ramoplanin A2 (**96**) prompt interest as it can overcome the resistance developed by some gram-positive bacteria to antibiotics based on glycopeptides, macrolides, and penicillins, but it is not orally absorbed and showed mild to moderate hemolytic efficacy when administered intravenously.

**Figure 12:**
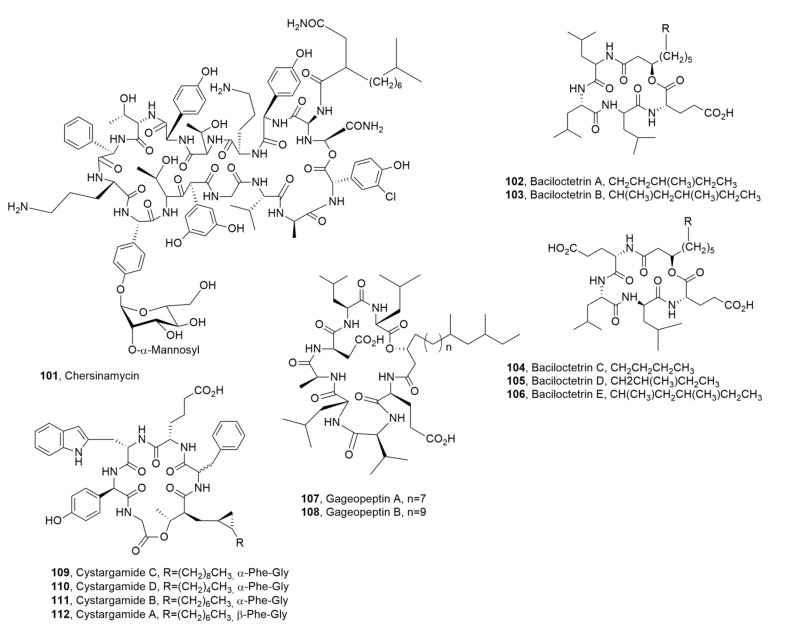
Structures of lipodepsipeptides produced by *Micromonospora chersina* (**101**), *Bacillus subtilis* (**102**–**108**), and *Streptomyces* sp. (**109**–**112**).

In addition, when dispensed by intraperitoneal injection its macrolactone is easily hydrolyzed [114]. Enduracidins A and, B, which had similar activity, but showed decreased water solubility, were approved in the United States only as a growth-promoting feed additive for livestock [115,116]. Ramoplanins and enduracinins were also produced by *Micromonospora chersina* strain DSM 44151, *Actinoplanes orientalis* strain DSM 40040, and *Actinoplanes balhimycina* FH 1894 strain DSM 44591, but only *M. chersina* produced in few amounts of the LPD named chersinamycin (**101**, Figure 12) [113].

Baciloctetrins A and B (**102** and **103**, Figure 12) were produced by *B. subtilis* isolated from a marine sponge sample collected from the Gageo reef, Republic of Korea. The antibiotic activity of LPDs **102** and **103** was evaluated against clinically isolated MRSA strains (ATCC25923, XU212, SA1199B, RN4220, and EMRSA15) [117]. Successively, three LPDs, named bacilotetrins C-E (**104**–**106**, Figure 12), were isolated from the same bacterium, which showed antibiotic activity against *Mycoplasma hyorhinis*. The latter is the main causal agent of polyserositis and arthritis in swine and is a common contaminant in laboratories. Compounds **104**–**106** showed activity, with an MIC value of 31 µg/mL, which was two folds stronger than that of the positive control, BioMycoX^®^. The same author also revised the structure of baciloctetrins A and B (**102** and **103**) [118]. Previously, two other LPDs, named gageopeptins A and B (**107** and **108**, Figure 12) were isolated from the same strain of *B. subtitlis*. LPDs **107** and **108** were able to impair the motility of zoospores of *Phytophthora capsici* in dose- and time-dependent manners. LPDs **107** and **108** also showed moderate antibacterial and good antifungal activities [119].

Cystargamides C and D (**109**–**110**, Figure 12) were isolated together with the already known cystargamide B (**111**, Figure 12) [72] from a marine actinomycete mixture of *Streptomyces* sp. (98.8% identical to *Streptomyces malachitofuscus*), which was collected at Beolgyo, South Korea. The LPDs **109**–**111** showed an antioxidant effect in the DPPH (2,2-diphenyl-1-picrylhydrazyl) and ABTS (2,2-azino-bis (3-ethylbenzothiazoline-6-sulfonic acid) radical-scavenging assay [73]. Previously, cystargamide A (**112**, Figure 12) had been isolated from *Kitasatospora cistarginea*. LPD **112** differed from cystargamide B for the stereochemistry of pheylglicine residue [120]. 

## 3. Source, Isolation, and Biological Activity of Fungal Lipodepsipeptides

Although the LPDs produced by fungi are less extended in respect to those ones synthesized by bacteria as reported in Section 2, they are interesting due to their chemical and biological properties.

They are chronologically described in this section with the exception of the case of LPDs belonging to the same subgroup.

**Figure 13:**
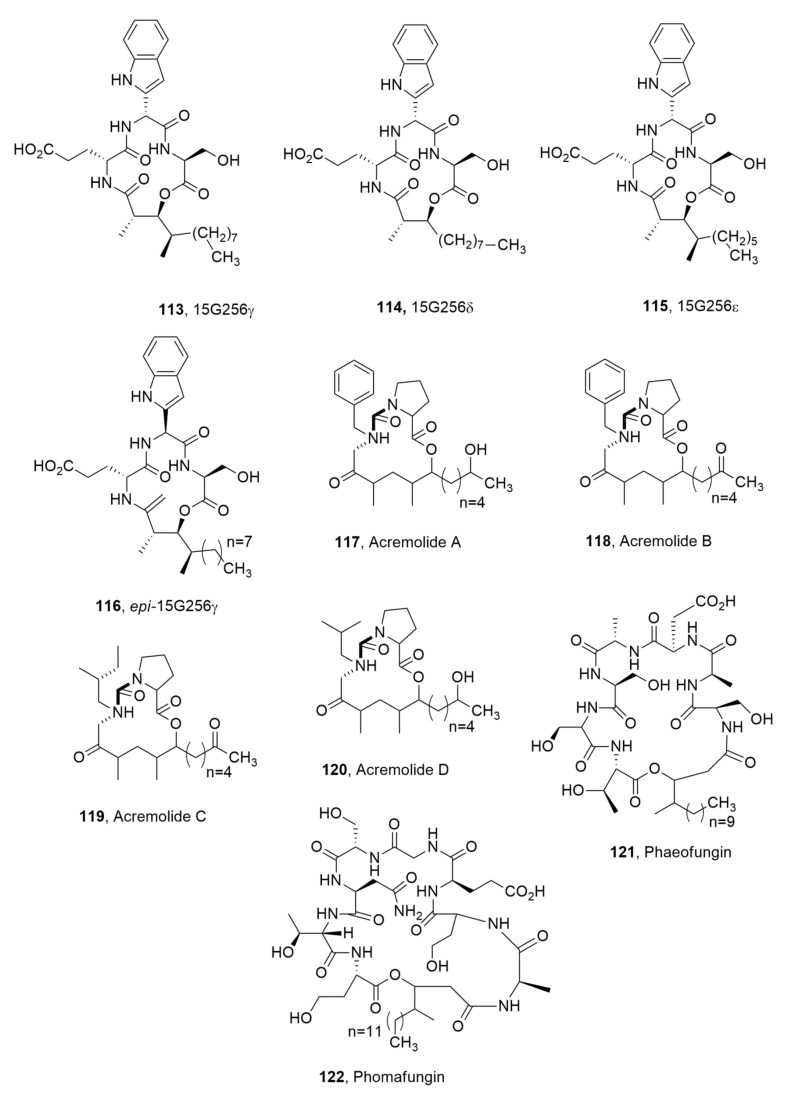
Lipodepsipeptides produced by *Hypxylon oceanicum* (**113**–**115**), *Acremonium* sp. (**117**–**120**), *Phaeoshaeria* sp. (**121**) and *Phoma* sp. (**122**).

The lipodepsipeptides 15G256γ, 15G256δ and 15G256ε (**113**–**115**, Figure 13) were isolated together with some polylactones from the marine fungus *Hypoxylon oceanicum*. Their structures are characterized by the presence of the unusual ketotryptophan amino acid, which is responsible for the epimerization occurring in solutions of the α-carbon, as occurring in the conversion of **113** in its epimer (**116**, Figure 13). LPDs **113**–**115** showed antifungal activity with compound **113** being the stronger one [121]. 

A novel family of LPDs, named acremolides A-D (**117**–**120**, Figure 13), were isolated together with the known 19-*O*-acetylchaetoglobosin D, 19-O-acetylchaetoglobosin B and aromatic metabolite RKB 3564S from an Australian estuarine strain of an *Acremonium* sp. (MST-MF588a). Among all of these, the two chetoglobusins are the only compounds showing cytotoxic properties [122]. The acremolides did not show cytotoxic activity but they were not tested for synergic activity [123].

Phaeofungin (**121**, Figure 13) was isolated from *Phaeosphaeria* sp. (F-167,953) obtained from the stems and leaves of a *Sedum* sp. (*Crassulaceae*), which was collected in Motilleja, Albacete, Spain. Moreover, a second strain (F-262,327, E-000531145) of the same fungus producing phaeofungin was isolated from the stems and leaves of *Teucrium* sp. (Lamiaceae) which was also collected in Spain but at Serrania de Cuenca, Cuenca. The whole fungal extract showed activity against wild-type *C. albicans* and *Aspergillus fumigatus*, two important human pathogens [124]. LPD **121** is very close to phomafungin (**122**, Figure 13) produced by *Phoma* sp. which was isolated from soil collected in Séréhini, Grand Comore, Union of the Comoros and showed a broad spectrum of antifungal activity against *Candida* spp., *A. fumigatus* and *Trichophyton mentagrophytes* [125]. LPD **121** induced ATP release in wild type of *C. albicans* strains, while LPD **122** did not [124]. 

**Figure 14:**
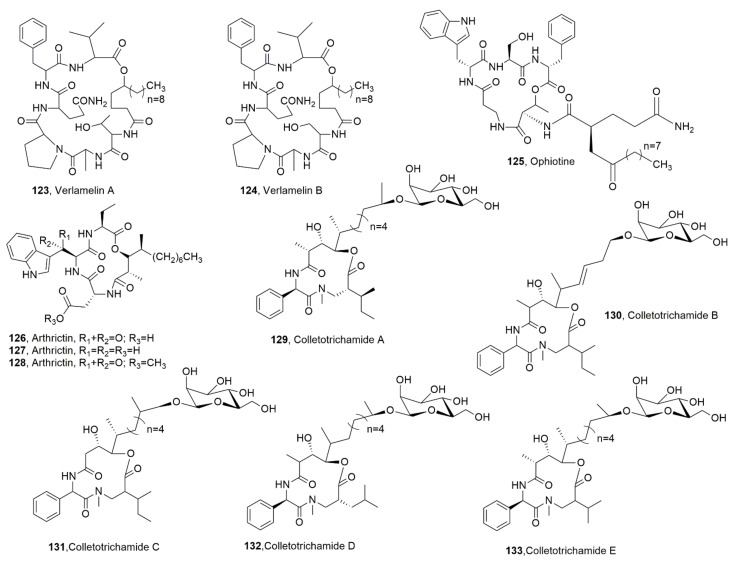
Lipodepsipeptide produced by *Lecanicillium* sp. (**123** and **124**), *Ophiosphaerella* genus (**125**–**128**), and *Colletotrichum gloesporioides* (**129**–**133**).

Verlamelins A and B (**123** and **124**, Figure 14) were isolated from the entomopathogenic fungus *Lecanicillium* sp. obtained from a chilli thrips cadaver. Both LPDs **123** and **124** showed antifungal activity against *Cochliobolus miyabeanus* and *Alternaria solani*, while verlamelin B was less active than the others against *Fusarium oxysporum*, *Cladosporium cucumerinum* and *Ustilago maydis* thus suggesting an important role played by the methyl group on the first amino-acid residue connected to the fatty acid moiety [126].

Ophiotine (**125**, Figure 14), was isolated, together with artthrichitin, arthrichitins B and C (**126**–**128**, Figure 14) and xanthomide Z from a fungus obtained from cysts of the nematode *Heterodera filipjevi*, which showed affinities to the genus *Ophiosphaerella*. LPD **125** showed moderate nematicidal activity against the host nematode, while xanthomide Z exhibited very weak activity. Arthrichitin C (**126**) showed weak cytotoxicity against several cancer cell lines [127].

Colletotrichamides A-E (**129**–**133**, Figure 14) were isolated from *Colletotrichum gloeosporioides* JS419, which was obtained from *Suaeda japonica* Makino. The latter was collected from a swamp in Suncheon, South Korea. All the LPDS **129**–**133** were tested for their protective activity on HT22 hippocampal cell death induced by glutamate. Compounds **130**, **131**, and **133** were found to be active, with LPD **128** being the most active metabolite [128].

**Figure 15:**
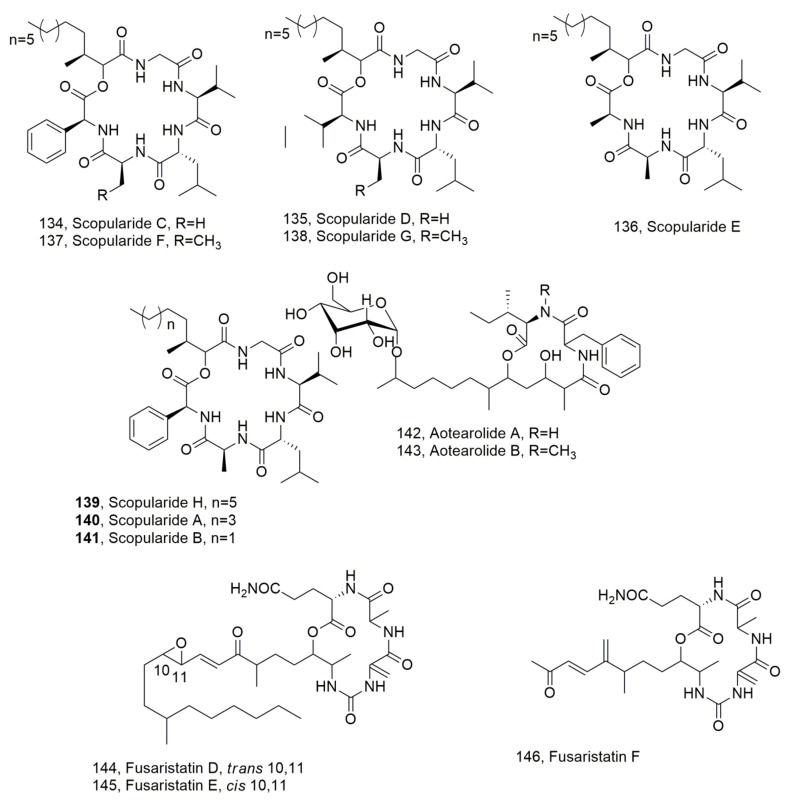
Structure of lipodepsipediteds produced by *Beauvaria* sp. (**134**–**138**), *Scopulariopsis* sp. (**139**–**141**), *Colletotrichum aotearoa* (**142** and **143**), and *Fusarium* sp. (**144**–**146**).

Scopularides C-G (**134**–**138**, Figure 15) were isolated from *Beauvaria* sp. CMB F585, while scopularide H (**139**, Figure 15) was obtained from *Scopulariopsis* sp. CMB F115 together with the already known scopularides A and B (**140** and **141**, Figure 15) [129]. All the strains were obtained from the gastrointestinal tract of Mugil mullet fish. LPDs **137** and **138** were also previously isolated from *Scopulariopsis brevicalis*. When tested for antibiotic and cytotoxic activity all LPDs were inactive [130].

Aotearolides A and B (**142** and **143**, Figure 15) were isolated together with the known 1*H*-indole-3-carbohaldehyde and 2-(1*H*-indole-3-yl)acetic acid from *Colletotrichum aotearoa*, which is an endophytic fungus of *Huperzia serrata* [131].

**Figure 16:**
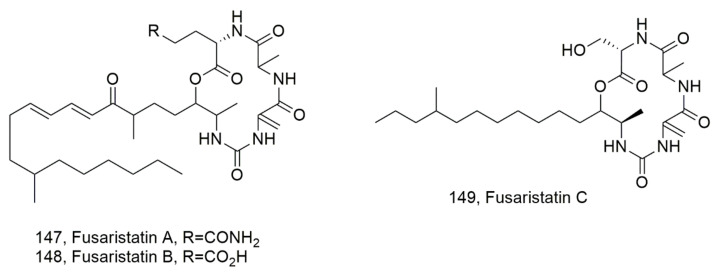
Structure of lipodepsipeptide produced by *Fusarium* sp. (**147** and **148**) and *Pithomyces* sp. (**149**).

Fusaristatins D-F (**144**–**146**, Figure 15), were isolated together with (-)-chlamydospore and eight known compounds from *Fusarium* sp. BZCB-CA, which is an endophytic fungus collected from the Chinese plant *Bothriospermum chinesis*. They did not show biological activity [132]. Previously, fusaristatins A and B (**147** and **148**, Figure 16) were isolated from *Fusarium* sp. YG collected from *Maackia chinensis* and showed cytotoxic activity against lung cancer cells [133]. More recently, fusaristatin C (**149**, Figure 16) was isolated from *Pithomyces* sp. collected from Caribbean octoral *Eunica fusca* and did not show antimicrobial or cytotoxic activity [134].

## 4. Lipodepsipeptides Produced by Bacteria Pathogens of Mushrooms

Some species of pathogenic bacteria are responsible for severe diseases in edible mushrooms such as brown blotch in *Agaricus bisporus* and yellowing of *Pleurotus ostreatum* induced by *P. tolaasii* [135]. Other bacteria are involved in *A. bisporus* and *Pleurotus* sp. diseases including *Pleurotus gyngerii*, which is cultivated in the Southern Italy [136,137]. *Burkholderia gladioli* pv. *agaricicola* is responsible for cavity diseases in white button mushrooms [138]. All these bacteria are able to produce bioactive LPDs showing phytotoxic as well as other interesting biological activities. *Pseudomonas gladioli* pv. *agaricicola*, *P. tolaasii* and *P. reactans*, which are all pathogens for cultivated mushrooms, produce lipodepsipeptides with different biological activities. 

**Figure 17:**
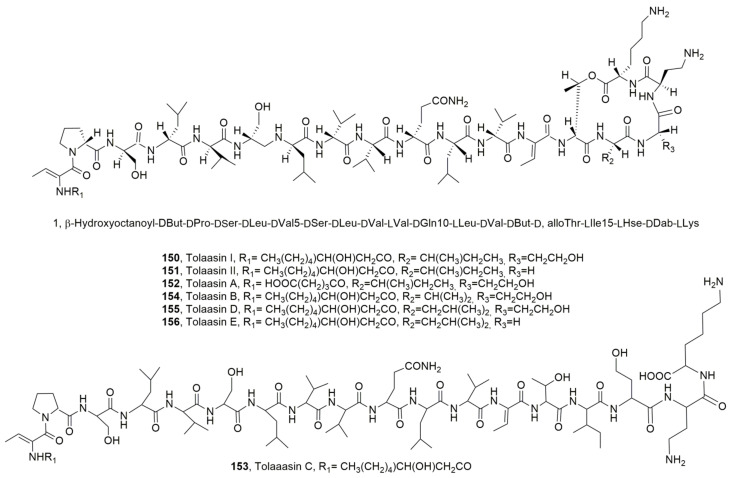
Structure of lipodepsipeptides produced by *Pseudomonas tolaasii* (**150**–**156**).

The main bioactive lipodepsipeptides produced by *P. tolaasii* are tolaasins I and II (**150** and **151**, Figure 17), whose structure was determined by Nutkins et al. (1991) [139]. LPDs **150** and **151** themselves differed in the substitution of homoserine residue (Hse16) of macrocyclic lactone with a glycine residue. Successively from the same bacterium four other close LPDs, named tolaasins A, B, D and E (**152**, **154**, **155** and **156**, Figure 17) were isolated [140]. Although tolaasins A, B, D and E showed the same macrocyclic lactone ring, they showed differences in the peptide moiety and maintained the same β-hydroxyocatnoyl ϕ chain at the *N*-terminus except for tolaasin A, in which the acyl moiety is a γ-carboxybutanoyl ϕ residue. In addition, tolaasin C (**154**, Figure 17) was also isolated and showed the opening of the macrocyclic ring while the peptide sequence was the same as tolaasins I. Thus, it could be generated by hydrolysis of LPD **150**. All the LPDs **150**–**153**, **155** and **156** showed antimicrobial activity against fungi, bacteria and yeasts [140].

**Figure 18:**
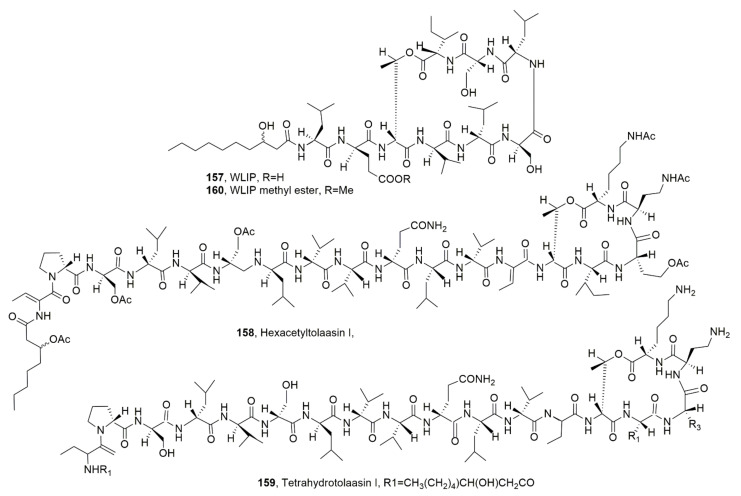
Structure of WLIP produced by *Pseudomonas reactans* and its methyl ester (**157** and **160**) and the hexacetyl- and tetrahydro-derivative of tolaasiin I (**158** and **159**).

*P. reactans* produced as the main LPD according to the so-called White Line Inducing Principle (WLIP, **157**, Figure 18), which in respect to tolaasins showed a shorter peptide side chain [50].

Tolaasin I and WLIP were compared for their different biological activity. Both LPD **150** and **157** inhibited the growth of mushrooms including *A. bisporus*, *Letinus edodes* and *Pleurotus* spp.-chromista, as well as that of gram-positive bacteria. LPD **150** showed antimicrobial activity against gram-negative bacteria including *Escherichia*, *Erwinia*, *Agrobacterium*, *Pseudomonas* and *Xanthomas* and antimicrobial activity against mushrooms, while WLIP affected only *Erwinia carotova* subp. *carotova*. Both LPDs induced hemolysis of red blood cells with a strongest effect exhibited by WLIP [137]. Both LPDs also affected the lipid membrane inducing calcein release from large unicellular vesicles [141].

Considering the food importance of mushroom species damaged by the above cited *Pseudomonas* strains and the consequent heavy economic losses, recently some attempts were made to avoid these severe problems. Some positive results were obtained using helper bacteria belonging to the *Mycetocola* genus which inactivated tolaasin by hydrolyzing the lactone ring and thus generating a liner peptide [142]. A different mechanism of tolaasin detoxification seemed to be operated by *Microbacterium foliorum*, which hydrolyzed the peptide chain in two points [143].

Tolaasins I, II, A, D and E, the hexacetyl- and tetrahydro-derivatives of tolaasin I, WLIP, its methyl ester and some cyclic dipeptides were assayed for their antimicrobial activity against bacteria and fungi pathogens for some important agrarian plants such as *Pseudomonas caryophilly*, *Pseudomonas syringae* pv. *panici*, *Pseudomonas syringae* pv. *tabaci*, *P. syringae* pv. *syrinage*, *Pseudomonas syringae* pv. *japonica*, *B. subtilis*, *B. megaterium*, *E. coli* and *Colletotrichum truncatum*. Among the LPDs the strongest antimicrobial activity was shown by tolaasin D [144].

## 5. Structure–Activity Relationship of SAR Studies

In this section the results of SAR studies carried out with some selected LPD groups from different sources and with different activities were reported.

In comparison with tolaasin I and II (**150** and **151**), Tolaasins A-E (**152**–**156**) when assayed against the gram-positive bacteria *B. megaterium* and *Rodococcus fascians*, the yeast *R. pilimanae*, the gram-negative bacteria *E. coli* and *E. c.* sups. *carotovorasis* and the fungus *Rizoctonia solani* inhibited the growth of the bacteria, except for tolaasin C, although differences among their specific activities were observed. Furthermore, tolaasin D was the most effective while tolaasins I and II were the least effective. Among the microorganisms tested, *B. megaterium* and *R. fascians* were the most sensitive to tolaasins A/B and E. Similar results were observed when testing all the LPDs against the fungus *R. solani*. The gram-negative bacteria were unaffected while the growth of *R. pilimanae*, that was less sensitive in respect to bacteria, was inhibited only by tolaasins I, II, and D. The results suggested the importance of both the lactone and the *N*-terminus acyl moiety. In fact, tolaasin A, which has pentanedioic acid instead of α-hydroxyoctanoic acid, and tolaasin C, which is a linear peptide, were inactive and less active in respect to LPD **150**, respectively. An aspect that also appeared to be important was the nature of the amino acid at position 15 as the substitution of isoleucine with a valine or leucine residue in tolaasins B and D, respectively, compared to the parent LPD **150**, induced a decrease or an increase of the antimicrobial, respectively. Similarly, the presence of leucine in position 15 in tolaasin E, in respect to LPD **151**, determined the reduction in the activity. This last result must be considered additionally taking into account the structural differences between LPDs **151** and **150** consistent in the presence of a glycine residue instead of homoserine at position 16 [140]. More recently, as reported above, tolaasins I, II, A, D and E, the hexacetyl- and tetrahydro-derivatives of tolaasin I (**158** and **159**, Figure 18), WLIP, its methyl ester (**160**, Figure 18) and some cyclic dipeptides were assayed for their antimicrobial activity against bacteria and fungi pathogens for some important agrarian plants. Among the LPDs and derivatives tested, only LPDs **150**, **151** and **155** and the tetrahydrotolaasin I inhibited all the bacteria and the fungus tested, while *E. coli* growth was not inhibited. Tolaasin E (**156**) and the hexacetyltolaasin I did not show activity against *B. subtilis*, *B. megaterium*, and *E. coli* but inhibited all the pathogenic bacteria and *C. truncatum*. The highest antimicrobial activity was shown by tolaasin D (**155**) while the less toxic ones appeared to be tolaasin E (**156**) and the two derivatives of tolaasin I [144]. These results showed that the amino acid residue at position 16 is not important for the antimicrobial activity as the L-homoserine present in LPD **150** was substituted by and L-serine in LPD **151** and both compounds have a similar antimicrobial activity. L-Hse is also present at the same position in tolaasin D (**155**). Thus, the increased activity shown by the latter LPD, with respect to the parents LPDs **150** and **151**, could be due to the presence of a different amino acid residue at position 15, which is L-leucine (L-Leu) in LPD **155** and L-isoleucine in the other two LPDS. However, the presence in the same lipodepsipeptide of L-Leu and L-Ser at positions 15 and 16, as observed in LPD **156**, induces a noteworthy decrease in antimicrobial activity. The acetylation of the hydroxyl group of both macrolactone and the petide chain as well as the hydrogenation of some residues of the latter determined a marked reduction of the activity [144]. In comparison ti LPDs **150**, **151** and **155**, WLIP (**157**), which differs from tolaasins for all the three moieties such as the fatty acid, the linear side peptide chain and the macrocyclic lactone, did not inhibit the growth of all pathogenic bacteria, but was active against gram-positive strains *B. subtilis* and *B. megaterium* and exhibited antifungal activity against *C. truncatum*. Similar activity was shown by WLIP methyl ester, suggesting that the ester group could probably be hydrolyzed under the physiological conditions [144].

As reported above *P. s.* pv. *syringae* synthesized both small cyclic lipodepsinonapeptides such as the syringomycins (**1**–**3**) and the larger cyclic LPDs syringopeptins SP22 or SP25 (**4** and **5**). The first LPD group inhibit a broad spectrum of fungi, but particularly yeasts through lipid-dependent membrane interaction while the others showed essentially phytotoxicity and inhibition of gram-positive bacteria. LPDs SP22A and SP25A, compared to LPD **1**, were less effective in inhibiting *Saccharomyces cerevisiae* and *C. albicans*. The same differences were observed for the ability to cause cellular K^+^ and Ca^2+^ fluxes in *S. cerevisiae*. Furthermore, syringopeptins were able to form larger single channels in the target yeast plasma membrane but using the same mode of action of syringomycin E. Thus, the difference in efficacy to inhibit the yeasts could be attributed to their different hydrophobicity, with SP22A and SP25A being more hydrophobic than syringomicin E and consequently, they interact more strongly with the yeast cell wall [145].

The viscosin included groups of LPDs produced by *Pseudomonas* bacteria showing a range of biological activities. Their oligopeptide moieties are composed of both L- and D-amino acids. The only residue present in both L or D configuration is Leu. The D/L switch has a determinantal impact on the LPD conformation in solution and consequently on the SAR results. On the basis of Leu configuration, the viscosins were divided into two subgroups, the L and the D ones. In some specific subgroups such as massetolides (L-Leu5) and the pseudophomins (D-Leu5), another variation of configuration needs to be considered and regards the isoleucine at position 4, which are D-allo-Ile4 and D-Ile4, respectively. The impact of the D-Leu5/L-Leu5 variation among the viscosin (**30**) LPD group was also studied deeply using viscosamide (**29**), a closely related minor metabolite. In fact, the structures in solutions of pseudodesmin A (**27**) and viscosinamide A (**29**) were calculated, showing that the overall peptide fold remains the same, but the surface distribution of the hydrophobic side chains, and thus amphipathicity, appeared affected. Consequently, the D/L switch appeared to be a tool to modulate the biosurfactant properties and biological function of this LPD group [146].

Among the different LPDs belonging to the tripropeptins group, that are reported above as A-E and Z, tripropeptin C (TPPC), which is the main component, showed potent antibiotic activity against a variety of different gram-positive pathogens, including MRSA strains, vancomycin-resistant *Enterococcus faecalis/faecium* (VRE) and penicillin-resistant *Streptococcus pneumoniae*. Furthermore, TPPC also showed a good toxicological profile. TPPC inhibited peptidoglycan biosynthesis in a different way than the drugs that currently target peptidoglycan biosynthesis, including vancomycin and bacitracin and was thus proposed as a potential new class of antibiotic against MRSA strains and vancomycin-resistant *E. faecalis/faecium*. On this basis, a SAR study focused on finding derivatives with increased antibiotic activity and/or selectivity was prompted. Thus, several derivatives of TPPC were hemisynthesized by chemical modification such as that of the two carboxyl groups. In particular, the carboxyl groups were firstly converted in the corresponding methyl ester and later by reduction into the corresponding primary alcohols. The carboxyl groups were also converted into the corresponding amides using amino acids which had different chain lengths. All the derivatives were assayed, in comparison to TPPC, against 10 clinical Methicillin-sensitive *S. aureus* (MSSA) and 10 clinical Methicillin-resistant *S. aureus* (MRSA) strains. Unfortunately, all of the modifications completed yielded derivatives with a decreased antibacterial activity in respect to that of TPPC suggesting the importance of the presence of the two free carboxyl groups to impart the antibiotic activity [147].

**Table 1:** Lipodepsipeptides isolated from bacterial and fungi in the last 3 decades.

Lpodepsipeptide	Source	Biological Activity	References
**Lipodepsipeptides produced by bacteria**			
Syringomicyn E (**1**)	*Pseudomonas syrungae* pv. *syringae*	Phytotoxic, Antifungal	[21]
Syringomicyn G (**2**)	“	“	“
Syringomicyn A_1_ (**3**)	“	“	“
Syringopeptin SP25A (**4**)	“	Phytotoxic	[4]
Syringopeptin SP25B (**5**)	“	“	“
Syringotoxin (**6**)	“	No Activity	”
Syringostatin A (**7**)	*Pseudomonas syringae* pv. *syringae* SY12	Phytotoxic	[25]
Syringostatin B (**8**)	“	“	“
Syrimgomicin (**9**)	*Pseudomonas syringae* pv. *syringae* SC1	Antimicrobial	[26]
Pseudomycin A (**10**)	*Pseudomonas syringae* MSU 16H	Phytotoxic, Antifungal	[27,29]
Pseudomycin B (**11**)	“	No Activity	[27]
Pseudomycin C (**12**)	“	“	“
Pseudomycin C’ (**13**)	“	“	“
Fuscopeptin A (**14**)	*Pseudomonas fuscovaginae*	Phytotoxic, Antifungal	[31]
Fuscopeptin B (**15**)	“	“	“
Syringopeptin (**16**)	*Pseudomonas syringae* pv. *syringae*	Phytotoxic	[7]
Syringopeptin SP22A (**17**)	“	Phytotoxic, Cytotoxic	[33]
Syringopeptin SP22B (**18**)	“	“	“
Corpeptins A (**19**)	*Pesudomonas corrugata*	Phytotoxiv, Antibiotic	[35]
Corpeptins B (**20**)	“	“	
Putisolvin I (**21**)	*Pseudomonas putida*	Antibiofilm	[37]
Putisolvin II (**22**)	“	“	“
Cormycin A (**23**)	*Pseudomonas corrugata*	Phytotoxic, Antibiotic, Red-blood-cell lysis	[36]
Syringopeptin 508A (**24**)	*P. syringae* pv. *lachrymans*	Antibiotic	[39]
Syringopeptin 508B (**25**)	“	“	“
LPDs A54145 (**26**)	*Streptomyces fradiae*	“	[40,41]
Pseudodesmin A (**27**)	*Pseudomonas tolaassii*	“	[44]
Pseudodesmin A (**28**)	“	“	“
Viscosamide (**29**)	*Pseudomonas fluorescens*	Biosurfactant, Antibiotic	[47]
Viscosin (**30**)	*Pseudomonas viscosa,* *Pseudomonas fluorescens* *Pseudomonas libanensis*	Antibiotic Phytotoxic, Surfactant Surfactant, Anticancer	[45] [51] [52]
Massetolide A (**31**)	*Pseudomonas*	Antibiotic	[53]
Massetolide B (**32**)	“	No Activity	“
Massetolide C (**33**)	“	“	“
Massetolide D (**34**)	“	“	“
Massetolide E (**35**)	“	“	“
Massetolide F (**36**)	“	“	“
Massetolide G (**37**)	“	“	“
Massetolide H (**38**)	“	“	“
Massetolide I (**39**)	“	“	“
Massetolide J (**40**)	“	“	“
Massetolide K (**41**)	“	“	“
Pseudophomin A (**42**)	*Pseudomonas fluorescens*	Phytotoxic, Antifungal	[49]
Pseudophomin B (**43**)	“	“	“
PPZPM-1a (**44**)	*Pseudomonas* sp. *JX090307*	No activity	[55]
PPZPM-2a (**45**)	“	“	“
Amphisin (**46**)	*Pseudomonas* sp.	Biosurfactant, Antifungal	[56] [57]
Cichopeptin A (**47**)	*Pseudomonas cichorii*	Phytotoxic, Antibiotic	[67]
Cichopeptin B (**48**)	“	“	“
Ralstonin A (**49**)	*Ralstonia solanacearum*	Chlamydospore-inducing activity, Phytotoxicity	[71]
Ralstonin B (**50**)	“	“	“
Cystargamide C (**51**)	*Streptomyces* sp.	Antioxidant	[73]
Cystargamide D (**52**)	“	“	“
Cystargamide B (**53**)	“	“	[72]
Stephensiolide A (**54**)	*Serratia* sp.	Antibiotic, Induction of bacterial motility	[74]
Stephensiolide B (**55**)	“	“	“
Stephensiolide C (**56**)	“	“	“
Stephensiolide D (**57**)	“	“	“
Stephensiolide E (**58**)	“	“	“
Stephensiolide F (**59**)	“	“	“
Stephensiolide G (**60**)	“	“	“
Stephensiolide H (**61**)	“	“	“
Stephensiolide I (**62**)	“	“	“
Stephensiolide J (**63**)	“	“	“
Stephensiolide K (**64**)	“	“	“
Fusaricidin A (**65**)	*Paenibacillus polymyxa*	Antimicrobial	[77]
Fusaricidin B (**66**)	“	“	[78]
Fusaricidin C (**67**)	“	“	“
Fusaricidin D (**68**)	“	“	“
Cichorinotoxin (**69**)	*Pseudomonas cichorii*	Phytotoxic	[85]
Lysocin E (**70**)	*Lysobater* sp.	Antibiotic	[86,89]
Lysocin I (**71**)	*Lysobacter enzymogenes*	“	[89]
Lysocin J (**72**)	“	“	“
Lysobactin (**73**)	*Lysobacter* sp.	“	[99,100]
Tripropetin A (**74**)	“	Antmicrobial	[92,93,101]
Tripropetin B (**75**)	“	“	“
Tripropetin C (**76**)	“	“	“
Tripropetin D (**77**)	“	“	“
Tripropetin E (**78**)	“	“	“
Tripropetin Z (**79**)	“	“	“
Tripropetin aiC (**80**)	“	“	[102]
WAP-8294A1 (**81**)	“ *Lysobacter antibioticus*	Antibiotic “	[104] [98]
WAP-8294A2 (**82**)	*Lysobacter* sp.	Antibiotic	[104]
WAP-8294A4 (**83**)	“	“	“
Isopedopeptin A (**84**)		Antibiotic	[105]
Isopedopeptin B (**85**)	*Pedobacter cryoconitis*	Antibiotic, Cytotoxic Hemolytic	“
Isopedopeptin C (**86**)	“	Antibiotic	“
Isopedopeptin D (**87**)	“	“	“
Isopedopeptin E (**88**)	“	“	“
Isopedopeptin F (**89**)	“	“	“
Isopedopeptin G (**90**)	“	“	“
Isopedopeptin H (**91**)	“	“	“
Bolagladin A (**92**)	*Burkholderia gladioli*	No activity	[106]
Bolagladin B (**93**)	“	“	“
Orfamide H (**94**)	*Pseudomonas protegens*	Antifungal	[108]
Ramoplanin A1 (**95**)	*Actinoplanes, Actinoplanes orientalis,* *Actinoplanes balhimycin,* *Micromonospora chersina*	Antibiotic	[112]
Ramoplanin A2 (**96**)	“	Antibiotic, Hemolytic	“
Ramoplanin A3 (**97**)	“	Antibotic	“
Ramoplanose (**98**)	“	“	“
Enduracidin A (**99**)	*Streptomyces fungicidicus, A. orientalis,* *A. balhimycin, M. chersina*	“	“
Enduracidin B (**100**)	“	“	“
Chersinamycin (**101**)	*Micromonospora chersina*	“	“
Baciloctetrin A (**102**)	*Bacillus subtilis*	“	[117]
Baciloctetrin B (**103**)	“	“	“
Baciloctetrin C (**104**)	“	Anti-micoplasma	[118]
Baciloctetrin D (**105**)	“	“	“
Baciloctetrin E (**106**)	“	“	“
Gageopeptin A (**107**)	“	Impaire fungal zoospore motility, Antibiotiv	[119]
Gageopeptin B (**108**)	“	“	“
Cystargamide C (**109**)	*Streptomyces* sp.	Antioxidant	[73]
Cystargamide D (**110**)	“	“	“
Cystargamide B (**111**)	“	“	[72,73]
**Lipodepsipeptides produced by fungi**			
Cystargamide A (**112**)	*Kitasatospora cistarginea*	No activity	[120]
15G256γ (**113**)	*Hypoxylon ocenicum*	Antifungal	[113]
15G256δ (**114**)	“	“	“
15G256ε (**115**)	“	“	“
*Epi*-15G256ε (**116**)	Chemical derivative of 113	No activity	“
Acremolide A (**117**)	*Acremonium* sp.	“	[123]
Acremolide B (**118**)	“	“	“
Acremolide C (**119**)	“	“	“
Acremolide D (**120**)	“	“	“
Phaeofungin (**121**)	*Phaeosphaeria* sp.	Antifungal	[124]
Phomafungin (**122**)	*Phoma* sp.	“	[125]
Verlamelin A (**123**)	*Lecanicillium* sp.	“	[126]
Verlamelin B (**124**)	“	“	“
Ophiotine (**125**)	Fungus of *Ophiosphaerella* genus	Nematocidal	[127]
Arthrichitin B (**126**)		No activity	“
Arthrichitin C (**127**)		Cytotoxic	“
Artrichitin (**128**)		No activity	“
Colletotrichamide A (**129**)	*Colletotrichum gleosporioides*	“	[128]
Colletotrichamide B (**130**)	“	Protective on HT22 cell hippocampal cell death	“
Colletotrichamide C (**131**)	“	“	“
Colletotrichamide D (**132**)	“	No activity	“
Colletotrichamide E (**133**)	“	Protective om HT22 cell hippocampal cell death	“
Scopularide C (**134**)	*Beauvaria* sp.	No activity	[130]
Scopularide D (**135**)	“	“	“
Scopularide E (**136**)	“	“	“
Scopularide F (**137**)	“	“	“
Scopularide G (**138**)	“	“	“
Scopularide H (**139**)	*Scopulariopsis* sp.	“	“
Scopularide A (**140**)	*Scopulariopsis brevicaulis*		[129]
Scopularide B (**141**)	“	“	
Aotearolide A (**142**)	*Colletotrichum aotearoa*	No activity	[131]
Aotearolide B (**143**)	“	“	“
Fusaristatin D (**144**)	*Fusarium* sp. BZCB-CA	“	[132]
Fusaristatin E (**145**)	“	“	“
Fusaristatin F (**146**)	“	“	“
Fusaristatin A (**147**)	*Fusarium* sp. YG-45	Cytotoxic	[133]
Fusaristatin B (**148**)	“	“	“
Fusaristatin C (**149**)	*Pithomyces* sp.	No activity	[134]
**Lipodepsipeptides produced by bacteria pathogen of mushrooms**			
Tolaasiin I (**150**)	*Psudomonas tolaasii*	Phytotoxic, Antimicrobial Hemolytic	[139] [140] [144] [137]
Tolaasiin II (**151**)	“	“	“
Tolaasiin A (**152**)	“	Antimicrobial	[140,144]
Tolaasiin B (**153**)	“	“	[140]
Tolaasiin C (**154**)	“	“	[140,144]
Tolaasiin D (**155**)	“	“	“
Tolaasiin E (**156**)			“
WLIP (**157**)	*Pseudomonas reactans*	Phytotoxic, Antimicrobial, Hemolytic	[50] [137,145] [139]

## 6. Conclusions

The review reports on the lipodepsipeptides isolated from bacteria and fungi including pathogens and endophytes. The different sources, chemical structures and biological activities of LPDs were described in detail. The differences between the different groups of LPDs and between those belonging to the same one were also highlighted in terms of structural features and biological activity. A separate section was organized to describe the LPDs isolated from *Pseudomonas* sp. pathogens for mushrooms which showed severe disease damage induced on their hosts and the consequent heavy economical losses. The same procedure was also carried out when LPDs were produced as phytotoxins produced by bacteria and fungi pathogens for important agrarian plants. The biological activity that LPDs showed against some human diseases was also described as well as the study carried out by synthesizing derivatives to perform SAR studies focused on increased activity and/or selectivity, as was the case in regard to antimicrobial activity. The potential practical application of some LPDs in medicine was also highlighted.

## Data Availability

Not applicable.
